# Fractionated Ionizing Radiation Promotes Epithelial-Mesenchymal Transition in Human Esophageal Cancer Cells through PTEN Deficiency-Mediated Akt Activation

**DOI:** 10.1371/journal.pone.0126149

**Published:** 2015-05-22

**Authors:** Enhui He, Fei Pan, Guangchao Li, Jingjing Li

**Affiliations:** 1 Nankai University School of Medicine, Tianjin, China; 2 Chinese PLA General Hospital and Chinese PLA Medical School, Beijing, China; 3 School of Bioscience and Bioengineering, South China University of Technology, Guangzhou, Guangdong, China; 4 Beijing Friendship Hospital, affiliated with Capital Medical University, Beijing, China

## Abstract

In some esophageal cancer patients, radiotherapy may not prevent distant metastasis thus resulting in poor survival. The underlying mechanism of metastasis in these patients is not well established. In this study, we have demonstrated that ionizing radiation may induce epithelial-mesenchymal transition (EMT) accompanied with increased cell migration and invasion, through downregulation of phosphatase and tensin homolog (PTEN), and activation of Akt/GSK-3β/Snail signaling. We developed a radioresistant (RR) esophageal squamous cancer cell line, KYSE-150/RR, by fractionated ionizing radiation (IR) treatment, and confirmed its radioresistance using a clonogenic survival assay. We found that the KYSE-150/RR cell line displayed typical morphological and molecular characteristics of EMT. In comparison to the parental cells, KYSE-150/RR cells showed an increase in post-IR colony survival, migration, and invasiveness. Furthermore, a decrease in PTEN in KYSE-150/RR cells was observed. We postulated that over-expression of PTEN may induce mesenchymal-epithelial transition in KYSE-150/RR cells and restore IR-induced increase of cell migration. Mechanistically, fractionated IR inhibits expression of PTEN, which leads to activation of Akt/GSK-3β signaling and is associated with the elevated levels of Snail protein, a transcription factor involved in EMT. Correspondingly, treatment with LY294002, a phosphatidylinositol-3-kinase inhibitor, mimicked PTEN overexpression effect in KYSE-150/RR cells, further suggesting a role for the Akt/GSK-3β/Snail signaling in effects mediated through PTEN. Together, these results strongly suggest that fractionated IR-mediated EMT in KYSE-150/RR cells is through PTEN-dependent pathways, highlighting a direct proinvasive effect of radiation treatment on tumor cells.

## Introduction

Esophageal cancer is one of the most challenging cancers to treat with the eighth highest mortality rate amongst all cancers worldwide.[1] It is the fourth most frequently diagnosed cancer and the fourth leading cause of cancer death in China.[2] Esophageal squamous cell carcinoma (ESCC) is the major histopathological subtype of esophageal cancer in China. Radiotherapy is the mainstay of the treatment of ESCC, but local failure has remained a major concern, with persistent or recurrent disease being reported in about 40–60% of patients.[3] A subset of esophageal cancer patients fail to respond to radiotherapy due to emergence of radioresistant (RR) tumor cells. The clinical course in these patients is characterized by frequent relapses and distant metastatic lesions. Investigating the underlying mechanisms involved in the development of RR tumor cells is of prime importance for studying the effect of radiotherapy on ESCC.

Epithelial–mesenchymal transition (EMT) is a process by which differentiated epithelial cells undergo remarkable morphological changes from an epithelial cobblestone phenotype to an elongated fibroblastic phenotype[3], which is characterized by decreased expression of epithelial markers such as E-cadherin and increased expression of mesenchymal markers such as vimentin and N-cadherin.[4] Currently, EMT has been implicated in two of the most important processes responsible for cancer-related mortality i.e. invasion and progression to distant metastatic disease, and acquisition of therapeutic resistance.[5] Recent studies suggest that EMT plays a crucial role in the development of cancer radioresistance. Radiation-mediated EMT has been widely studied in various types of tumors, both *in vitro* and *in vivo*, such as colorectal, cervical, breast and lung cancer.[6–10] However, the precise role of EMT and the underlying mechanisms involved in the development of radioresistance in esophageal cancer remains to be elucidated.

Phosphatase and tensin homolog (PTEN) is an important tumor-suppressor gene, which is a negative regulator of the phosphatidylinositol-3-kinase (PI3K)-Akt pathway, thereby participating in the regulation of the cell cycle, proliferation, apoptosis, cell adhesion, and EMT during embryonic development and cancer progression.[11, 12]. It has been reported that the decreased expression of PTEN, post-IR, induces radioresistance through promotion of cell proliferation and inhibition of cell apoptosis in non-small cell lung cancer and nasopharyngeal carcinoma.[13, 14] Directly restoring PTEN function has previously been reported to be a valuable approach to achieve radiosensitization *in vitro*.[15] However, whether PTEN participates in radiation-triggered EMT and tumor metastasis in ESCCs is not yet clear.

Therefore, in the present study, we hypothesized the role of PTEN in the development of radiation-induced EMT in ESCC. We examined if (i) irradiation could enhance the invasiveness of ESCCs by inducing EMT, (ii) PTEN deficiency was involved in radiation-induced EMT, and (iii) radiation-induced EMT was through activation of Akt/GSK-3β/Snail signaling.

## Materials and Methods

### Materials

Roswell Park Memorial Institute (RPMI) 1640 medium was obtained from Gibco (Life Technologies Corporation, IN), Fetal bovine serum (FBS) was purchased from HyClone (Thermo, MA). Antibodies for PTEN, E-cadherin, N-cadherin, Slug, Snail, vimentin, Akt, phosphorylated Akt (p-Akt), GSK-3β, phosphorylatedGSK-3β(p-GSK-3β) and horseradish peroxidase (HRP)-conjugated secondary antibody were purchased from CST (Cell Signaling Technology, MA). The PI3K inhibitor, LY294002, was purchased from Sigma (Sigma-Aldrich, MO). GoScript Reverse Transcription System and GoTaq qPCR Master Mix Kit were from Promega (Promega, WI).

### Cell line and cell culture

Human ESCC cell line, KYSE-150 (JCRB1095, provided by Dr. Fangfang Bu)[16, 17], was cultured in RPMI 1640 medium with 10% FBS and 100 units of penicillin-streptomycin and incubated at 37°C with 5% CO_2_ in a humidified incubator. Before culture, KYSE-150 cell line was authenticated by short tandem repeat profiling (Beijing Microread Gene Technology Co., China).

### Fractionated ionizing radiation (FIR) treatment [18]

The KYSE-150 cells were cultured in 25 cm^2^ flasks. On attaining approximately 75% confluence, the cells were irradiated with 1 Gy of X-ray at room temperature using the Hitachi MBR-1520-R irradiator (Hitachi Medical Corporation, Tokyo, Japan) at a dose rate of 4.73Gy/min, and the cells were returned to the incubator immediately after irradiation. Cells were sub-cultured into new flasks at approximately 90% confluence. This process was repeated so as to achieve exposure to radiation at a dose of 1 Gy, 3 times; 2 Gy, 3 times; and 4 Gy, 7 times, and every exposure was at an interval of three days; with the total dose being 37 Gy over a 2-month period. Several clones were isolated from the RR cells and individually cultured. The parental cells were treated using the same procedure except that they were not irradiated. Clonogenic assays were used to determine the resistance level and a RR clone named KYSE-150/RR was selected for our experiment.

### Clonogenic survival assay

Parental and RR cells were plated on six-well plates at 500, 1000, 2000, 3000, 4000 cells per well. Twenty four hours later, the cells were treated with an individual dose of radiation (0, 2, 4, 6, or 8Gy respectively). Cells were returned to the incubator immediately after irradiation to allow colonies to form. After 14 days, the colonies were fixed with methanol and stained with crystal violet. Colonies containing >50 cells were counted under the microscope. Surviving fraction, (SF) was calculated as the Number of Colonies / (Cells Seeded x Plating Efficiency). A survival curve was derived using GraphPad Prism 5.0. Each experiment was repeated at least three times.

### Plasmid construction and transfection

Recombinant plasmid pcDNA3.0-PTEN was constructed by the insertion of human PTEN cDNA into pcDNA3.0 vector (Invitrogen, CA) with primers as follows: forward, 5’ cggggtaccccggccaccATGACAGCCATCATCAAAGAGATC 3’ (underlined Kpn1+kozak) and reverse, 5’ ccgctcgagcggTCAGACTTTTGTAATTTGTGTATGC 3’ (underlined Xho1). KYSE-150/RR cells were transfected with pcDNA3.0-PTEN or pcDNA3.0 vector control. All transfections were carried out using ScreenFectA Transfection reagent (InCellA, Egggenstein-Leopoldshafen, Germany) as per manufacturer’s instructions. Transfected cells were analyzed by Western blotting, or quantitative reverse transcription–polymerase chain reaction (RT-qPCR).

### RNA interference assay

KYSE-150 cells, which were not irradiated, were transfected with 100 nmol of small interfering RNA (siRNA)-PTEN or nontargeting scrambled-siRNA using ScreenFectA Transfection reagent as per manufacturer’s instructions. siRNA-PTEN was synthesized using the following sequence: 5’-GGGCCAGGTCATAAATAAT-3’; while the Scrambled-siRNA sequence was as follows: 5’-UUCUCCGAACGUGUCACGUTT-3’.

### Western blot analysis

Cells were lysed in sodium dodecyl sulfate (SDS) lysing buffer, and were homogenized for protein extraction. Equal amounts of protein from each lysate were separated by SDS-PAGE (10% acrylamide) and blotted to PVDF western blotting membranes (Millipore Corporation, MA). This was followed by incubation with primary antibodies to PTEN, E-cadherin, N-cadherin, Slug, Snail, vimentin, Akt, p-Akt and glyceraldehyde 3-phosphate dehydrogenase (GAPDH, as loading control), and HRP-conjugated secondary antibody. The blot signals were then visualized with enhanced chemiluminescence (Thermo, MA).

### RNA extraction and RT-qPCR assay

Total RNA from cultured cells, after various treatments, was extracted with TRIZOL reagent (Life Technologies Corporation, IN) according to the manufacturer’s instructions. One microgram of total RNA was used for reverse transcription. One microliter of the total reverse transcription product was added to real time-qPCR mixture (20 μL) containing 10 μL 2×GoTaq qPCR Master Mix and primers (sense and anti-sense; 1μL) to determine the mRNA expression of human PTEN, E-cadherin, N-cadherin, Snail, Slug, vimentin and β-actin. The gene expression of mRNA from each sample was calculated by normalizing with β-actin. All primers were designed using IDT PrimerQuest Input software (Table 1). Samples were amplified with a precycling hold at 95°C for 30 s, 40 cycles of annealing and extension at 60°C for 34 s. Each measurement was performed in triplicate with ApplidBiosystems 7500 StepOne Real-Time PCR System (Applied Biosystems, Inc., CA) and analyzed using Applied Biosystem 7500 software v2.0.1.

**Table 1:** Primers used for RT-qPCR.

Primer	Sequence (5'-3')
Homo sapiens E-cadherin	FW: CGAGAGCTACACGTTCACGG
	RV: GGGTGTCGAGGGAAAAATAGG
Homo sapiens N-cadherin	FW: TGCGGTACAGTGTAACTGGG
	RV: GAAACCGGGCTATCTGCTCG
Homo sapiens snail	FW: TCGGAAGCCTAACTACAGCGA
	RV: AGATGAGCATTGGCAGCGAG
Homo sapiens slug	FW: CGTCACGACGGGTCAGAT
	RV: ATCTGACCCGTCGTGACG
Homo sapiens vimentin	FW: AGTCCACTGAGTACCGGAGAC
	RV: CATTTCACGCATCTGGCGTTC
Homo sapiens PTEN	FW: AGAAAGCTTACAGTTGGGCCCTGT
	RV: GCCACAGCAAAGAATGGTGATGCT
Homo sapiens β-actin	FW: CGGGAAATCGTCCGTGACATTAAG
	RV: TGATCTCCTTCTGCATCCTGTCGG

### Migration and invasion assay

The migration assays were performed in a 24-well transwell chamber containing polycarbonate filters with 8-μm pores (Corning, Acton, MA). A total of 500 μL medium containing 20% FBS as the chemotactic factor was added to the lower compartment and then 1×10^5^ cells of each group in serum-free DMEM were added to the upper compartment of the chamber and incubated for 24 h. The upper surface of the chamber was then scraped to remove non-migratory cells. Migrated cells were stained with 0.1% crystal violet and photographed under a light microscope (×100). The chambers were washed with 100 μL of 10% acetic acid and the OD of the stained cell eluent was measured at 560 nm. Each group was prepared in three duplicate wells. For invasion assay, a transwell system coated with 2 mg/mL of basement membrane, matrigel, (Corning, Acton, MA) was used. The rest of the procedure was similar to the migration assay as described above.

### Wound-healing assay

Cells were seeded in 12-well plates and cultured until 40% confluence; the confluent cell monolayer was scratched with a 20 μL pipette tip to produce a vertical line across the middle of the wells. The spread of wound closure was observed after 0 and 24 h interval and was photographed using a light microscope.

### Statistical analysis

Differences were evaluated by Student’s t-test for comparison of two groups. Data were expressed as the mean ±standard deviation (SD). A *P* value of <0.05 was considered as statistically significant.

## Results

### Effect of irradiation on cellular morphology and EMT markers

**Fig 1:**
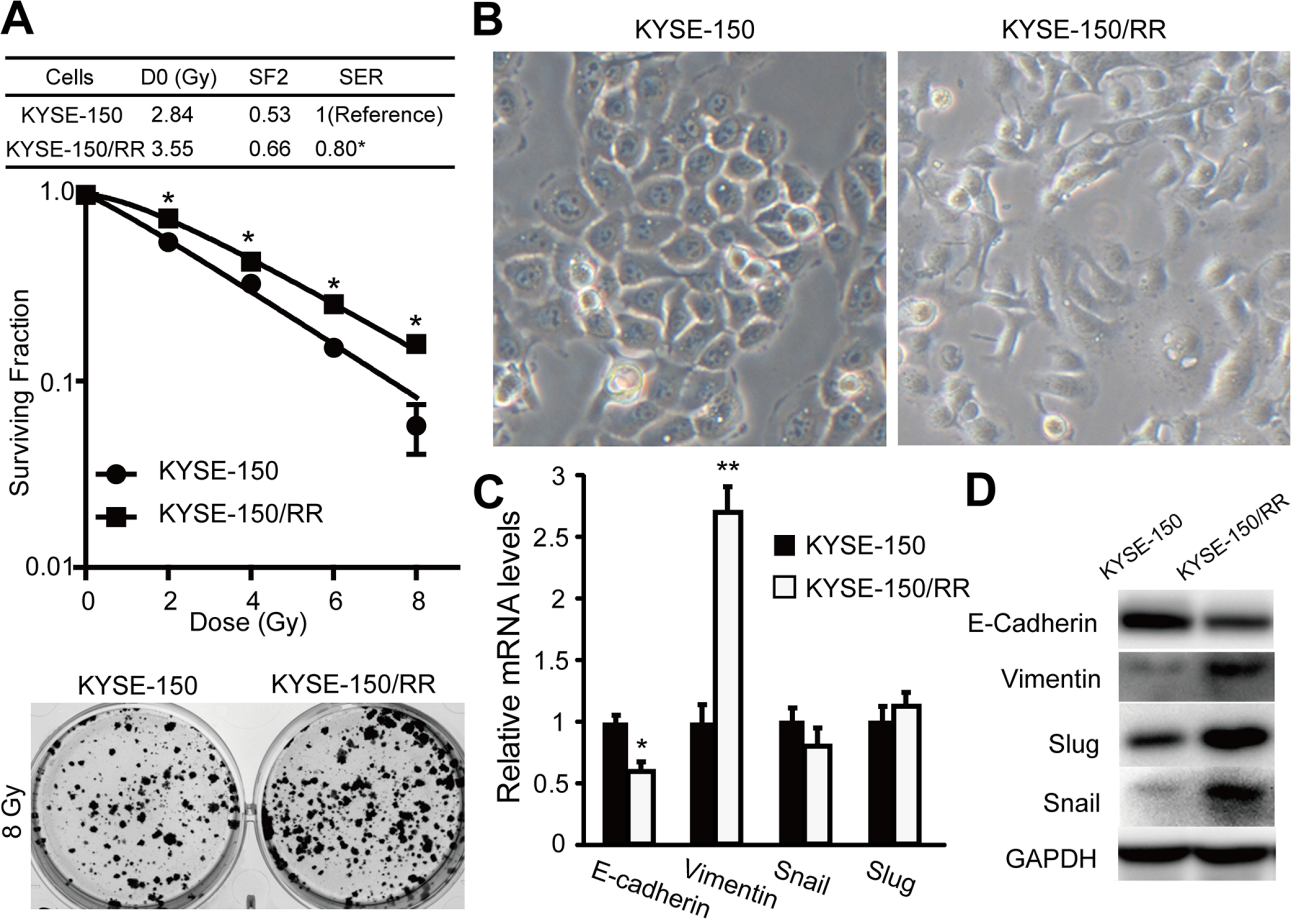
Irradiation induced molecular and phenotypic changes of EMT. (A) Radiation cell survival curves and the clonogenic figures of the control KYSE-150 cells without irradiation and radioresistance subclone KYSE-150/RR cells. (B) Morphology of KYSE-150 and KYSE-150/RR cells was examined with phase-contrast microscopy. (C) Expression of EMT markers (E-cadherin and vimentin) and transcription repressors of E-cadherin (Snail and Slug) were detected by qRT-PCR, data shown as mean ±SD, *P <0.05. Data represent means with standard deviation from three independent experiments. (D) Representative western blots of E-cadherin, vimentin, Snail and Slug were showed.

After two months of FIR with a total dose of 37 Gy, subclones were isolated and named KYSE-150/RR cells, and their RR character was demonstrated by clonogenic cell survival assay. Fig 1A shows that KYSE-150/RR cells survived for a longer period when compared to parental cells.

The RR cells demonstrated morphological changes. The control KYSE-150 cells (KYSE-150 Ctrl) had an epithelium-like morphology, with tight cell-cell conjunction and cobblestone-like appearance (Fig 1B left). The KYSE-150/RR cells developed a spindle-like morphology, with increased formation of pseudopodia and loss of cell-to-cell contact, which is characteristic of mesenchymal phenotype (Fig 1B right). The gain of these morphological features in RR sublines might hint towards its transformed characteristics, such as migration and invasion.[19]

To confirm whether this phenotype change was attributed to EMT, the mRNA and protein expression of EMT-associated genes were detected by qRT-PCR and Western blots. KYSE-150/RR cells showed the downregulation of epithelial marker E-cadherin, and upregulation of mesenchymal marker vimentin, when compared with KYSE-150 Ctrl cells (Fig 1C and 1D). Snail and Slug, negative regulators of E-cadherin, were critical for EMT.[19] In KYSE-150/RR cells, both Snail and Slug were significantly increased at the protein level (Fig 1D), but were not changed at the mRNA level (Fig 1C). These results demonstrate that irradiation is sufficient to induce EMT in ESCC cell line.

### Effects of irradiation on cell migration and invasion

**Fig 2:**
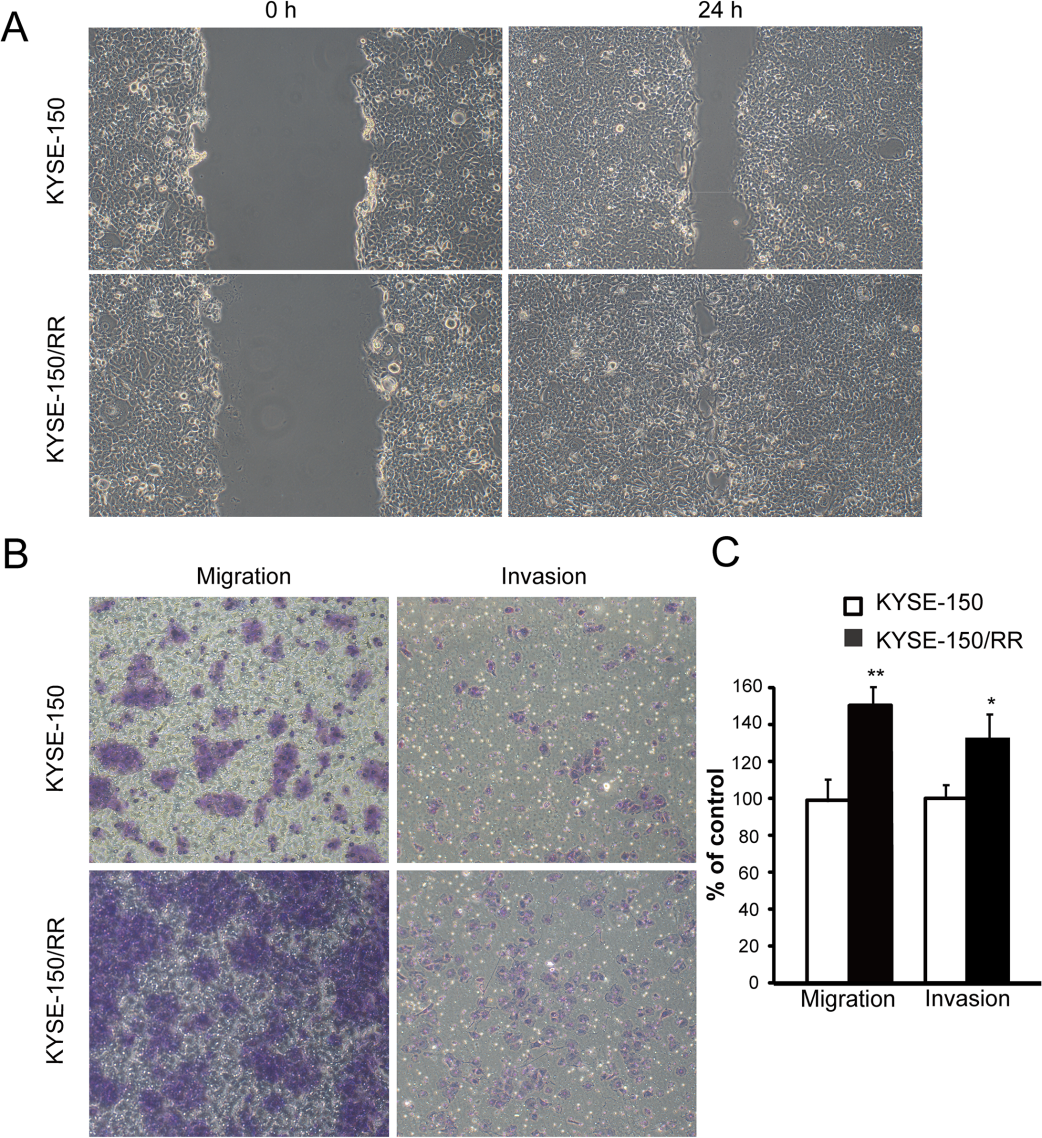
Irradiation-induced EMT enhanced cellular mobility. (A) KYSE-150 cells were subjected to a wound-healing assay with or without radiation at 100× magnification. Representative images were photographed right and 24 h after the scratch. (B) Representative images of migration assay and invasion assay of KYSE-150 cells with or without radiation were photographed after 24 h with crystal violet stain. (C) Summary graphs for migration and invasion (data shown as mean ±SD, * *P* <0.05).

Tumor cells that undergo EMT have increased cell migration and invasion ability. To investigate whether KYSE-150/RR cells display such traits, we evaluated the migration and invasion ability of KYSE-150 Ctrl and KYSE-150/RR cells *in vitro*. Wound-healing assay showed that KYSE-150/RR cells had significantly faster closure of wound area compared with KYSE-150 Ctrl cells (Fig 2A). Cell migration and invasion of KYSE-150/RR cells was found to be increased by 50% and 33% respectively, when compared with KYSE-150 Ctrl cells (Fig 2C). Representative images for the migration and invasion ability from each of the cell line are shown in Fig 2B.

### PTEN deficiency is required for irradiation-induced EMT

**Fig 3:**
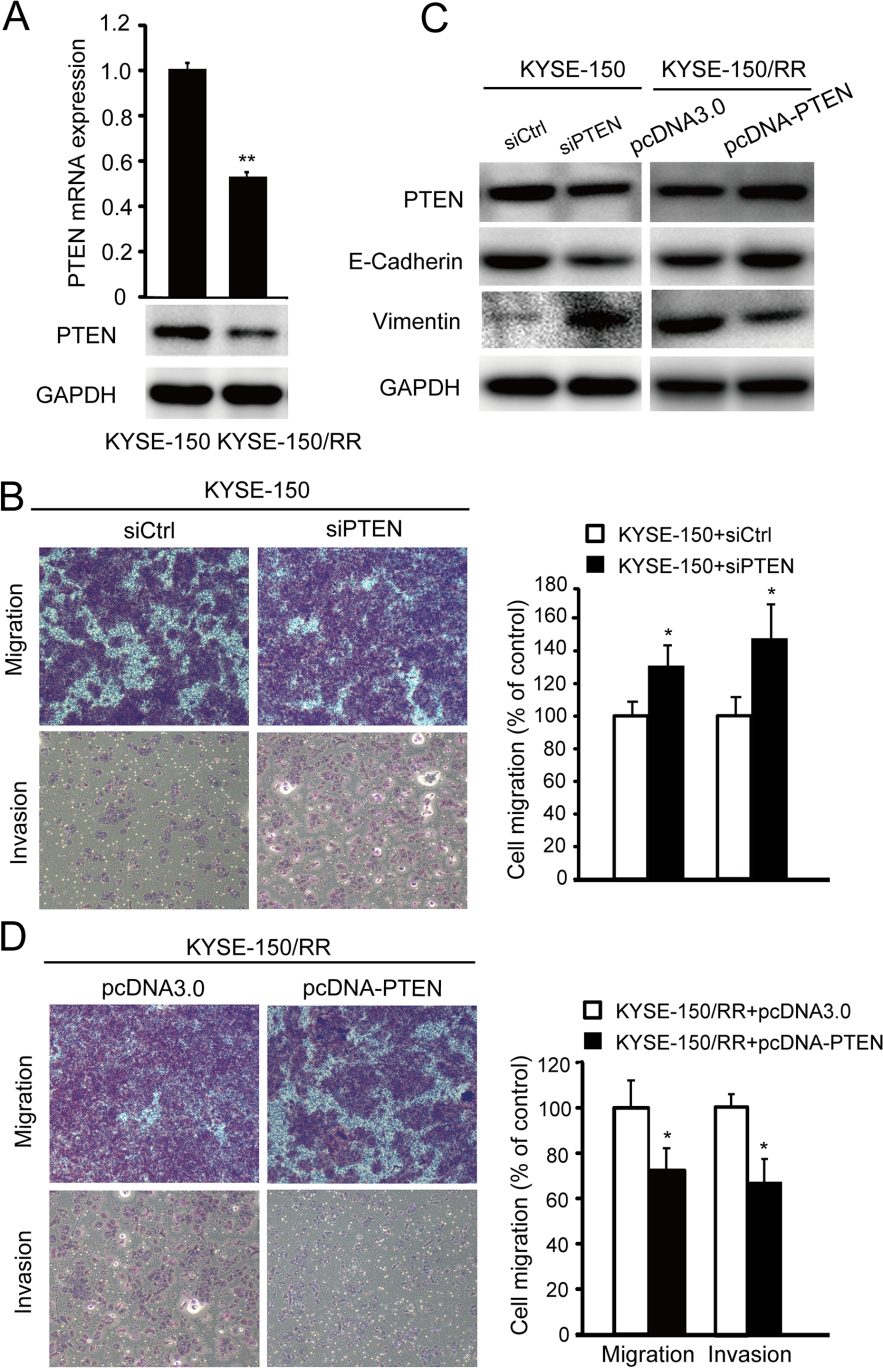
PTEN deficiency is required for irradiation-induced EMT. (A) Radiation significantly decreases the expression of PTEN in the KESE-150/RR cells no matter in mRNA level (data presented as mean ±SD, **P <0.01) and protein level (representative western blots). (B) The transfected efficiency of siPTEN in KYSE-150 (left) and pcDNA-PTEN in KYSE-150/RR cells (right) (data presented as mean ±SD, **P <0.01). (C) Representative western blot analysis showed expression of PTEN, E-cadherin and vimentin in KYSE-150 cells transfected with siPTEN or vehicle, or KYSE-150/RR cells transfected with pcDNA-PTEN or pcDNA3.0. Data shown represent three different experiments. (D) Representative images of migration and invasion assay of KYSE-150 cells transfected with siPTEN or vehicle after 48 h (left); Summary graphs is for migration and invasion (right, data shown as mean ±SD, *P <0.05). (E) Representative images of migration assay and invasion assay of KYSE-150/RR cells transfected with pcDNA-PTEN or pcDNA3.0 were photographed after 24 h with crystal violet stain (left); Summary graphs for migration and invasion (right, data shown as mean ±SD, *P <0.05). siPTEN is short for siRNA-PTEN.

It is reported that expression of PTEN is decreased in RR tumor cells.[13, 14] We investigated the mRNA and protein expression of PTEN in KYSE-150 Ctrl and KYSE-150/RR cells. Our results showed that radiation significantly decreased the expression of PTEN both at mRNA and protein levels (Fig 3A). To determine the effects of PTEN deficiency on cell migration and EMT, siRNA targeting of PTEN was used (Fig 3B left). Migration and invasion assay demonstrated that the knockdown of PTEN resulted in a clear migratory phenotype in KYSE-150 cells when compared with the vehicle transfected cells (Fig 3D). Western blots showed that KYSE-150-siPTEN cells exhibited loss of cell adhesion marker E-cadherin and elevation in mesenchymal differentiation marker vimentin (Fig 3C left).

We then used a vector encoding PTEN (Fig 3B right), to confirm the role of PTEN in radiation-induced EMT and tumor metastasis. We found that overexpression of PTEN caused KYSE-150/RR cells to restore the expression of E-cadherin and decrease the expression of vimentin (Fig 3C right). Moreover, the increased migration and invasion abilities of KYSE-150/RR cells were significantly inhibited by overexpression of PTEN (Fig 3E). The data indicates that the PTEN deficiency is necessary for radiation-induced EMT and migratory/invasive phenotype.

### PTEN decreased Snail expression through activation of PI3K/Akt/GSK-3β signaling

**Fig 4:**
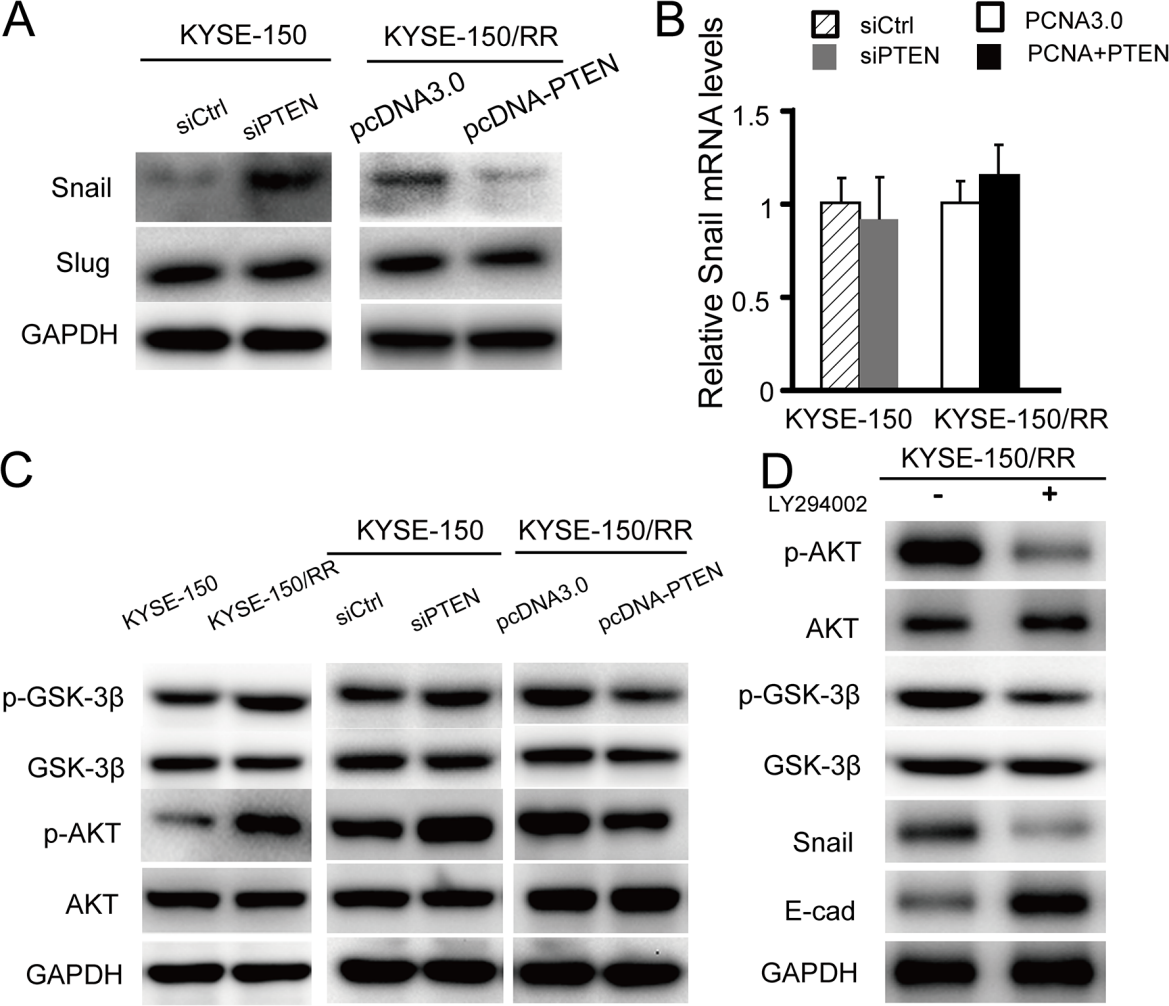
PTEN decreased Snail expression through inactivation of PI3K/Akt/GSK-3β signaling. (A) Expression of Snail and Slug detected by western blot analysis in KYSE-150 cells transfected with siPTEN or vehicle, or KYSE-150/RR cells transfected with pcDNA-PTEN or pcDNA3.0. (B) Expression of Snail were detected by qRT-PCR, data shown as mean±SD. (C) Representative western blot analysis showed expression of Akt, p-Akt, GSK-3β and p-GSK-3β. Data shown represent three different experiments. siPTEN is short for siRNA-PTEN. (D) Representative western blot analysis showed expression of Akt, p-Akt, GSK-3β, p-GSK-3β, Snail and E-cadherin in KYSE-150/RR cells with or without the phosphatidylinositol 3 kinase (PI3K) inhibitor, LY294002 (40 μM).

Snail and Slug are transcriptional repressors which play important roles in tumor metastasis by downregulating E-cadherin.[19] As mentioned earlier, Snail and Slug were upregulated in KYSE-150/RR cells at protein level, but not at mRNA level. To determine whether the elevated expression of Snail and Slug were due to PTEN deficiency upon irradiation, we changed PTEN expression by transfecting with siRNA against PTEN or PTEN expression vector, and then Western blots were performed to determine the expression of Snail and Slug. As shown in Fig 4A, Snail expression significantly increased in KYSE-150 Ctrl-siPTEN cells compared to KYSE-150 Ctrl-siCtrl cell, whereas, it was decreased in KYSE-150/RR-pcDNA-PTEN cells when compared to KYSE-150/RR-pcDNA cells. In contrast, expression of Slug was not significantly changed upon PTEN expression. Moreover, qRT-PCR showed no significant change in Snail mRNA levels (Fig 4B). These results indicate that the upregulation of Snail by irradiation is due to the decline in PTEN levels.

Activation of the PI3K/Akt/GSK-3β pathway is emerging as a central feature of EMT, with GSK-3β regulating the Snail expression through posttranslational modification.[20, 21] We hypothesize whether PTEN regulates Snail expression through PI3K/Akt/GSK-3β pathway in ESCC cells. As shown in Fig 4C, phosphorylation of Akt and GSK-3β was upregulated in KYSE-150/RR cells but not in KYSE-150 Ctrl cells. And the knockout of PTEN indeed facilitated the phosphorylation of Akt in KYSE-150 Ctrl cells, accompanied by an increase in phosphorylation of GSK-3β (Fig 4C, middle). In contrast, overexpression of PTEN inhibited Akt and GSK-3β phosphorylation in KYSE-150/RR cells (Fig 4C, right). To further provide proof of PTEN-dependent inhibition of PI3K/Akt/GSK-3β signaling, the PI3K inhibitor, LY294002, was used to mimic the effect of PTEN overexpression in KYSE-150/RR cells. LY294002 (40 μM) inhibited Akt and GSK-3β phosphorylation in KYSE-150/RR cells, and the expression of Snail was downregulated and that of E-cadherin protein was upregulated (Fig 4D). These results suggest that PTEN deficiency activates Akt/GSK-3β/Snail pathway upon irradiation.

## Discussion

Radiotherapy is an effective treatment for esophageal cancer even at an advanced stage. However, recurrence and metastasis due to development of radioresistance among tumor cells remains a major challenge. Previous studies have shown that RR phenotype was correlated with several factors, including alterations in cell cycle checkpoints, slowed growth, and decreased apoptosis.[22, 23] Epithelial mesenchymal transition is also thought to be regulated by exposure to radiation. Accumulated evidences indicate that ionizing radiation is one of the inducers of EMT, which has been shown to occur at the time of initiation of metastasis, leading to cancer progression, and is associated with the development of resistance to radiotherapy in oral, breast and lung cancers.[6, 7, 24]

To investigate clinical radioresistance, regimens of FIR have been used *in vitro* to determine molecular mechanisms underlying radioresistance. In this study, we developed KYSE-150/RR cells derived from clones that had survived after FIR treatment,[18] which appropriately mimics radiotherapy resistance in ESCC. KYSE-150 cell is a widely-used esophageal cancer cell line for the study of radiation resistance in esophageal carcinoma.[18, 25–27] KYSE-150 cell line was developed by Dr. Yutaka Shimada in 1991 from the poorly differentiated ESCC resected from the upper esophagus of a 49-year-old Japanese woman receiving radiotherapy.[16] Therefore, this cell line can be easily modified into a RR cell line. We found that the phenotype of KYSE-150/RR cells switched from an epithelial morphology to a mesenchymal morphology after irradiation. Simultaneously, we conducted migration and invasion assay and found that the migration and invasion ability of KYSE-150/RR cells was increased when compared with the parental cells, suggesting that EMT is involved in development of radioresistance and metastasis in ESCC.

A key step in EMT is downregulation of E-cadherin. E-cadherin is regulated either by transcription factors, such as Snail-related zinc-finger transcriptional repressors (Snail and Slug), SIP-1/ZEB-2 and basic helix–loop–helix transcription factor, Twist, or by ubiquitylation-induced endocytosis. In our study, we found FIR increased expression of both Snail and Slug at protein level and didn’t change their mRNA expression. Ectopic overexpression of Snail also leads to the acquisition of increased resistance to apoptosis and cancer stem cell-like properties in various epithelial cells.[28] The activation of Snail and Slug may be one of mechanisms involved in the development of radioresistance in ESCC after radiotherapy.

Activation of the protein kinase B (Akt) pathway plays a central role in the three major radioresistance mechanisms, which are intrinsic radioresistance; tumor-cell proliferation and hypoxia. PTEN, as an inhibitor of Akt, is reported to associate with radiosensitivity. It has been reported that the expression of PTEN was decreased in RR cells of gastric[15] and nasopharyngeal[14] carcinoma, meanwhile, others find PTEN to be essential for attenuation of invasion and EMT.[11, 29] We found the expression of PTEN to be downregulated after FIR, and overexpression of PTEN restored the phenotype of KYSE/150/RR cells from the mesenchymal morphology to the epithelial morphology, accompanied with decreased migration and invasion.

To further investigate the role of PTEN in radiation-triggered EMT, Akt/GSK-3β/Snail pathway was studied, which has been previously implicated in the development of radiation-induced lung cancer.[10, 30] We hypothesized a role for PTEN that leads to the increased E-cadherin expression via the Akt/GSK-3β/Snail pathway. Active GSK-3β can phosphorylate Snail to facilitate its degradation, [20, 21] conversely inactivation of GSK-3β, can stabilize Snail.[30] Our data showed that overexpression of PTEN can attenuate radiation-induced expression of Snail via dephosphorylation of the Akt in KYSE-150/RR cells, which accelerated the downregulation of p-GSK-3β and promoted Snail degradation. Snail, as one of transcription repressors of E-cadherin, upon getting downregulated caused the upregulation of E-cadherin expression. For further proving the involvement of PI3K/Akt/GSK-3β signaling in a PTEN dependent manner, PI3K inhibitor, LY294002, was used to mimic the effect of PTEN overexpression in KYSE-150/RR cells. LY294002 inhibited Akt and GSK-3β phosphorylation in KYSE-150/RR cells, and the expression of Snail was downregulated and E-cadherin protein was upregulated. Considered together, the data suggests that the downregulation of PTEN is linked with inactivation of GSK-3β, and PTEN-dependent PI3K/Akt/GSK-3β signaling is necessary to upregulate Snail for radiation-induced EMT to develop in ESCCs.

Vimentin, a major cytoskeletal component of mesenchymal cells, is often used as a marker of EMT during metastatic progression. It has been reported that blockage of PI3K/Akt signaling attenuated vimentin expression in oral carcinoma cells.[31] In our study, vimentin was up-regulated in KYSE-150/RR cells (Fig 1D) and overexpression of PTEN inactivated PI3K/Akt signaling leading to downregulation of vimentin expression in these cells (Fig 1C). This suggests that PTEN deficiency is required for radiation-induced EMT. It is noteworthy that Slug, another E-cadherin transcription repressor, is up-regulated in radiation (Fig 1D); however, Slug levels were not found to be affected by PTEN expression (Fig 4A). This finding indicates that radiation-induced EMT is a complex process and other pathways may also be involved.

**Fig 5:**
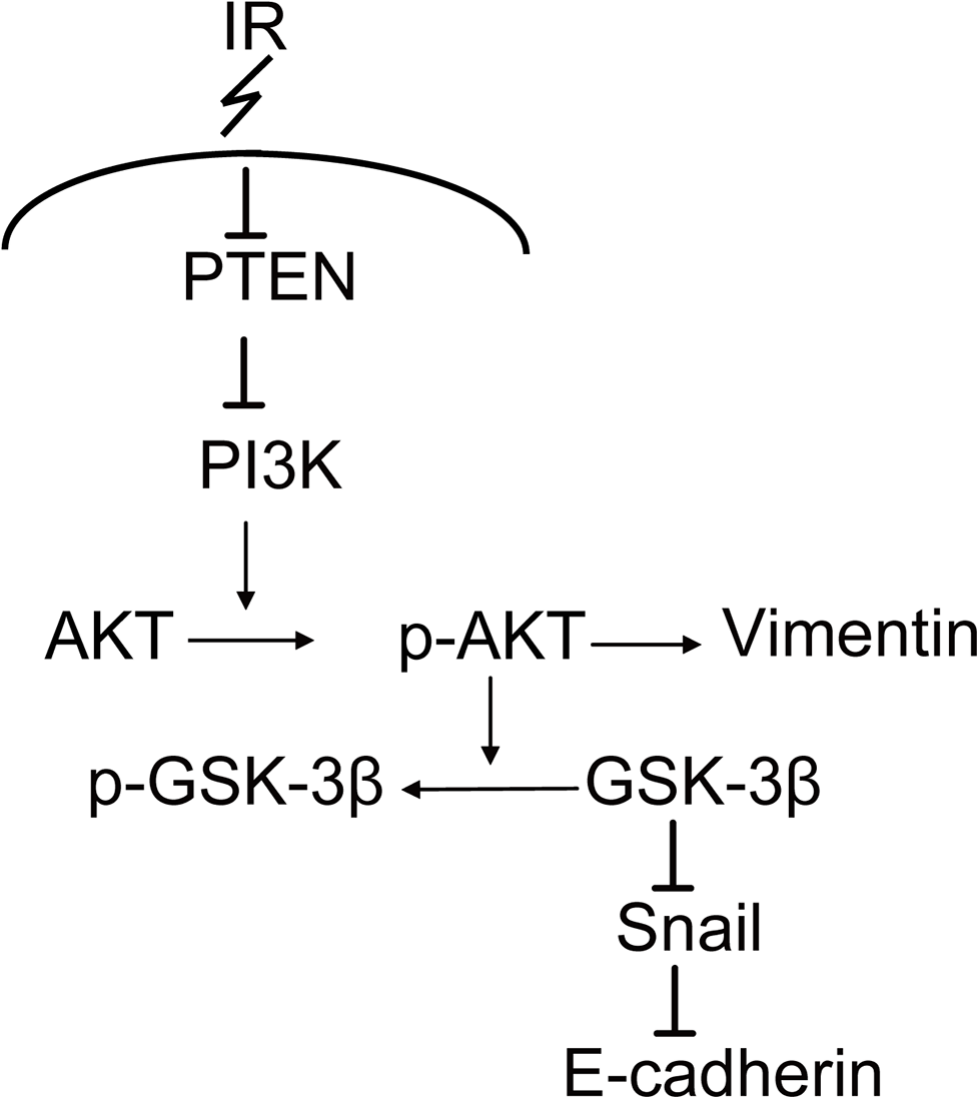
Proposed pathway for irradiation induced EMT. PTEN acts as an upstream regulator of the PI3K/Akt/GSK-3β signaling network to evoke the cascades of EMT. Slug regulates E-cadherin expression in a PTEN independent way.

Our study has elucidated the details of the EMT pathway in radiation triggered ESCC (Fig 5). This study highlights the importance of PTEN as a potential therapeutic target. The downregulation of PTEN and upregulation of the PI3K pathway triggered by radiation evokes the cascades leading to EMT. All these effects together contribute to tumor metastasis and recurrence after radiotherapy. Therefore, we suggest that PTEN deficiency subsequent to radiotherapy for esophageal cancer plays an important role in the development of RR tumor metastasis. Thus, overexpression of PTEN may prove to be a useful strategy to impede cancer cell invasion and metastasis that may develop subsequent to radiotherapy in ESCC.
