# Correction: Fractionated Ionizing Radiation Promotes Epithelial-Mesenchymal Transition in Human Esophageal Cancer Cells through PTEN Deficiency-Mediated Akt Activation

**DOI:** 10.1371/journal.pone.0133097

**Published:** 2015-07-14

**Authors:** Enhui He, Fei Pan, Guangchao Li, Jingjing Li

There are errors in the author affiliations. The affiliations should appear as shown here:

Enhui He^1,2,4^, Fei Pan^5^, Guangchao Li^3^, Jingjing Li^2,4^

1 Nankai University School of Medicine, Tianjin, China, 2 Chinese PLA General Hospital and Chinese PLA Medical School, Beijing, China, 3 School of Bioscience and Bioengineering, South China University of Technology, Guangzhou, Guangdong, China, 4 Beijing Friendship Hospital, affiliated with Capital Medical University, Beijing, China, 5 Department of Gastroenterology and Hepatology, Chinese PLA General Hospital and Chinese PLA Medical School, Beijing, China.
